# Association between rural clinical experience and rural career intentions amongst Slovenian medical students: a national cross-sectional study

**DOI:** 10.1186/s12909-026-10133-w

**Published:** 2026-08-11

**Authors:** Mirjam Capuder, Urban Bleiweis, Klara Gačnik, Veronika Vezjak, Marija Petek Šter, Ksenija Tušek Bunc

**Affiliations:** 1 Faculty of Medicine, University of Maribor, Taborska ulica 8, Maribor, 2000 Slovenia; 2 Faculty of Medicine, University of Ljubljana, Vrazov trg 2, 1000 Ljubljana, Slovenia

**Keywords:** Rural health, Medical students, Career choice, Medical education, Rural clinical experience, Family medicine, Slovenia

## Abstract

**Background:**

Rural medicine is fundamental to primary healthcare in Slovenia, where an estimated 60% of the population resides in rural areas. Despite rural family physician shortages, little is known about Slovenian medical students’ perceptions and interest in rural practice, factors shaping their career intentions and career choice toward rural medicine. Understanding these influences is essential for informing medical education and workforce policy aimed at strengthening healthcare provision in remote areas.

**Methods:**

A national cross-sectional survey was conducted amongst Slovenian medical students. A questionnaire assessing demographics, rural clinical experience, perceptions of rural practice, and career preferences was distributed to all enrolled students (*N* = 1,952). The questionnaire combined multiple choice, Likert scales and open-ended questions. Data were analysed using descriptive statistics, multivariate logistic regression, Wilcoxon rank-sum tests, and chi-square tests.

**Results:**

A total of 329 students completed the survey (response rate 19.1%), of whom 37.1% reported rural clinical experience. Students from the University of Ljubljana had substantially more rural clinical experience than those from the University of Maribor (46% vs. 14.9%). rural clinical experience was significantly associated with more positive perceptions of rural physicians and greater interest in working in rural areas (χ² *p* < 0.001), but not with interest in specialising in family medicine (χ² *p* = 0.182). Among students interested in rural work, family medicine was the most preferred specialty (42.1%). Students from a rural background had more than twice the odds of interest in family medicine specialty, compared to urban students (OR 2.19, 95% CI 1.31–3.71, *p* = 0.003). Financial incentives were the strongest motivating factor for rural career choice, selected by 72% of respondents.

**Conclusions:**

Rural clinical experience during medical education is positively associated with interest in rural practice and shapes students’ perceptions of rural medicine. Differences between the two faculties highlight the role of curriculum design in workforce preparation. Introducing structured rural placements and targeted financial incentive programmes may represent feasible strategies to address rural physician shortages.

**Graphical Abstract:**

**Figure:**
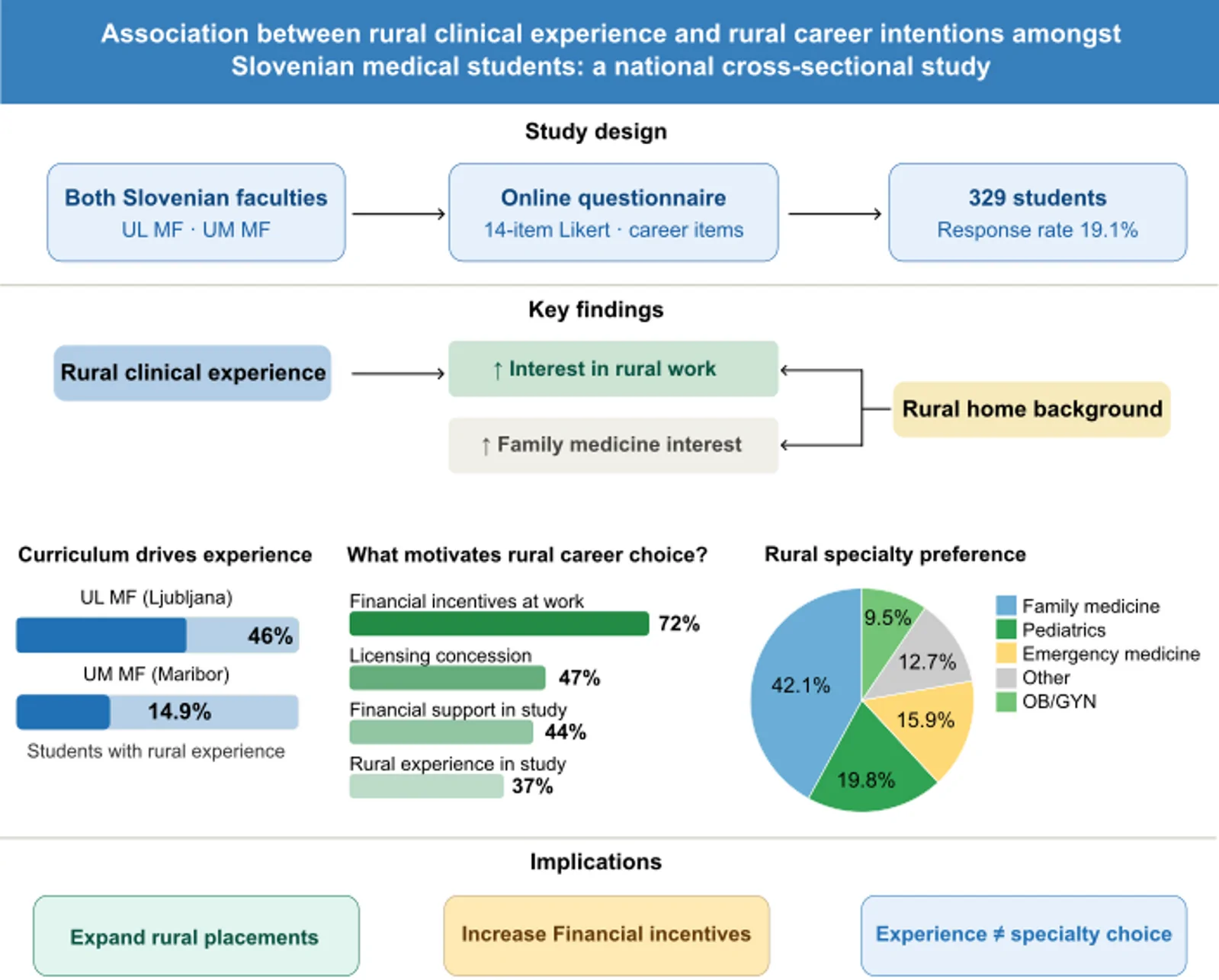

**Supplementary Information:**

The online version contains supplementary material available at https://doi.org/10.1186/s12909-026-10133-w.

## Background

**Fig. 1:**
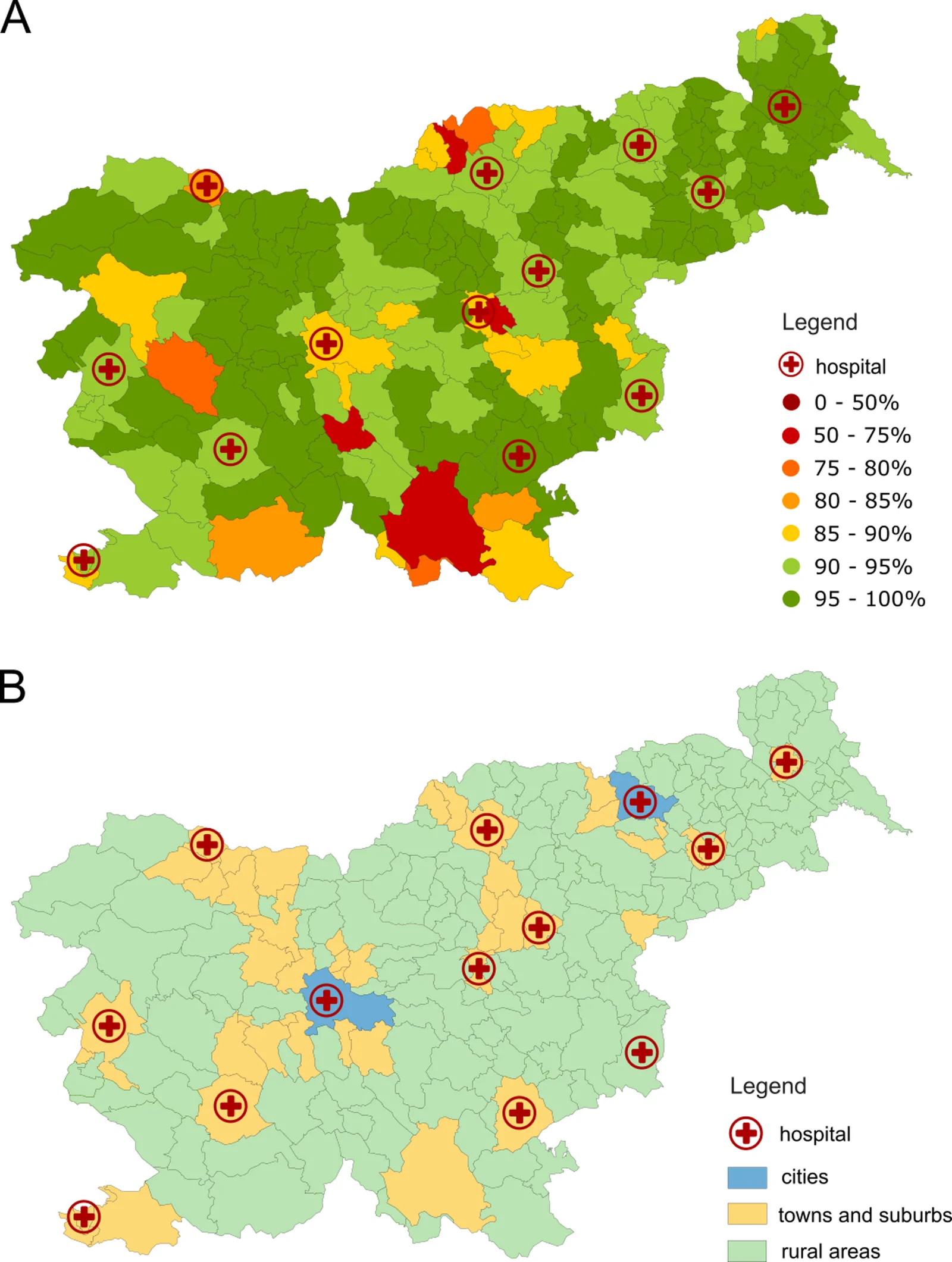
Demographic distribution of primary care physician shortage and rural–urban context in Slovenia. (**A**) Municipalities classified by the proportion (%) of insured individuals with an assigned personal physician. Red colour indicates low proportion/coverage, green colour indicates a higher proportion/coverage of insured individuals. (**B**) Rural–urban classification of municipalities, providing context of geographic disparities in access to primary care. Major hospitals are shown in both panels to support interpretation of spatial patterns. Data for panel A were obtained from publicly available analytics by Zdravniški Sledilnik, and data for panel B from the Statistical Office of the Republic of Slovenia [6, 10]

Defining rural medicine is inherently context‑dependent, and numerous country and situation-specific classifications exist due to differences in geographic, demographic, and infrastructural characteristics [1–3]. Given Slovenia’s relatively small geographic size, the definition of rural healthcare has specific contextual characteristics, including population size, distance to the nearest healthcare facility, and the degree of professional isolation [4]. Approximately 52.2% of Slovenia’s built-up surface, defined as the gross surface bounded by the building wall perimeter, is categorised as rural, with around 58% of the population, or 1.2 million residents living in these areas [5–8]. Only Ljubljana and Maribor qualify as urban centres, accounting for 17% of the built-up surface (Fig. 1) [6]. In Slovenia, rural and remote family practices are defined as those located in areas with fewer than 5,000 inhabitants situated at least 15 km from major cities, in very rural, agricultural, mountainous, or sparsely populated regions, or in remote areas more than 20 km from the nearest hospital or emergency unit [4]. Given that approximately 58% of Slovenia’s population resides in rural areas [9], ensuring adequate access to family medicine services is essential to support the health and well-being of these communities.

Rural recruitment programmes, designed to mitigate physician shortages in underserved areas, have focused on attracting new physicians to rural practice and retaining those already working in these settings [11]. Rural placements during medical education have been positively associated with later rural practice [12–14]. Given medical students’ interest in learning more about rural practice, innovative educational programmes have been developed that, incorporating real-life clinical narratives, which have been shown to positively influence perceptions of rural practice [15, 16]. Slovenia has two medical schools, Faculty of Medicine at the University of Ljubljana (UL MF) and Faculty of Medicine at the University of Maribor (UM MF), which graduate approximately 300 doctors annually. The 6-year curricula are broadly similar, with both programmes incorporating family medicine as a mandatory subject. UL MF has developed a dedicated elective in rural medicine, along with family medicine clinical placements in rural settings. In contrast, UM MF does not offer a dedicated rural medicine elective, but students can complete their family medicine clinical placements in rural settings. The elective course “Medicine in Rural and Remote Areas” at UL MF offers students direct experience to outpatient rural practice, combining reflective seminar work with supervised clinical placements in rural family medicine settings. The course aims to develop students’ understanding of the distinctive professional and organisational challenges of rural healthcare delivery. Such differences in curricular experience may shape students’ perceptions of rural practice and their interest in rural medical careers.

Family physician shortages in Slovenia are well documented and have worsened in recent years. As of 2025, an estimated 158,000 people are without a personal doctor [10, 17]. Municipal-level coverage varies considerably across the country (Fig. 1A), with shortages appearing in both selected urban centres and rural or border municipalities rather than following a simple urban–rural divide [10]. Given the need for access in rural areas, securing an adequate future workforce is essential. Identifying factors that effectively motivate medical students to consider a career in rural medicine can help policymakers, local authorities, and educational institutions to develop targeted programmes to strengthen the rural healthcare workforce.

Despite the importance of rural healthcare in Slovenia, no national study has yet examined Slovenian medical students’ perceptions of rural practice, their interest in rural careers, and the role of rural clinical experience in shaping these views. This study aimed to (1) assess medical students’ perceptions of rural practice, (2) evaluate their interest in rural careers, and (3) examine the association between rural clinical experience and career intentions.

## Methods

### Study aim, design and participants

This multicentre cross-sectional study included a national sample of medical students in Slovenia. Students from all years of the general medicine programme (Years 1–6 and gap-year students) at the Faculty of Medicine, University of Ljubljana (UL MF), and the Faculty of Medicine, University of Maribor (UM MF), were invited to participate.

Data were collected during a single defined study period (February 18th – April 18th 2025) using a structured questionnaire. The survey was distributed to all enrolled students at both medical faculties (*N* = 1,952; 1,297 in Ljubljana and 655 in Maribor). The overall response rate was 19.1%.

Inclusion criteria were: (1) current enrolment in the undergraduate medical programme (years 1–6 or gap-year status), (2) completion of the entire questionnaire and (3) provision of informed consent. Questionnaires that were incomplete or lacked informed consent were excluded from analysis.

Participation was voluntary and anonymous. No identifying information was collected. At the beginning of the questionnaire, participants were informed that proceeding with the survey indicated informed consent. Privacy and confidentiality were strictly maintained throughout the study.

### Study procedure

To inform questionnaire development, a qualitative descriptive analysis was performed on 63 seminar papers written by third- and fourth-year students at UL MF between the academic years 2020/21 and 2023/24 within the elective course “Medicine in Rural and Remote Areas.” The seminar papers contained reflective accounts of clinical experiences in rural outpatient settings and were used to identify recurring themes among medical students that were related to their understanding of rural medical practice. For each seminar paper, all key observations regarding the work of a doctor in rural practice were systematically extracted and recorded. The frequency with which specific student perspectives were identified across all 63 seminar papers was compiled into a summary table. To ensure consistency, conceptually similar and synonymous observations were grouped into common thematic categories. The categories that occurred most frequently were subsequently used to inform the development of the questionnaire items.

Based on the seminar analysis, a review of relevant literature, and consensus among the research team, a questionnaire to examine Slovenian medical students’ interest in rural practice, explore factors influencing their motivation to work in rural settings, and assess the effect of rural clinical placements on their interest in rural medicine was developed. Questions regarding participants’ demographic profiles were developed based on a review of prior literature linking demographics to specialty and practice-location choices [18–22]. The questionnaire was pilot tested on ten medical students from different study years at both faculties to evaluate clarity, terminology, structure, and estimated completion time and did not undergo formal psychometric validation.

Data were collected using an online questionnaire. The invitation link was disseminated through university email lists, social media platforms, student groups, and in-person announcements during lectures and practical sessions. The survey was open for three months and two reminder emails were sent during the data collection period. Collected data were cleaned and prepared for statistical analysis.

### Questionnaire

The final questionnaire consisted of 18 items divided into four sections, described below.

1. Demographic characteristics (Q1–Q8).
2. Experience with rural clinical practice (Q9–Q10).
3. Perceptions of characteristics of rural medical work (Q13).
4. Career preferences and motivational factors (Q12, Q14–Q18).

The questionnaire comprised four sections. The first section captured demographic characteristics, including gender, year of study, university, home area, and parental education level (Q1–Q8). The second section assessed prior experience with rural clinical practice, asking whether students had undertaken any rural clinical activity during their training and, if so, its approximate duration (Q9–Q10). The third section evaluated perceptions of rural medical work using 14 statements rated on a five-point Likert scale (1 = strongly disagree to 5 = strongly agree; Q13). Statements were formulated so that higher scores generally reflected a more accurate and positive understanding of rural medical practice. One item, addressing opportunities for professional consultation, was reverse-coded to detect inattentive responding. The fourth section addressed career preferences and motivational factors (Q11, Q14–Q18). Interest in specialising in family medicine and interest in working in a rural area were each assessed with a single item using a five-point Likert scale (1 = not interested at all to 5 = very interested). The likelihood of working in a rural setting in the future was assessed with the item “How likely are you to work in a rural setting in the future?” rated on a five-point scale (1 = very unlikely to 5 = very likely). Students were also asked to select, from a predefined list, any factors that would motivate them to pursue rural practice.

### Definition of variables

For statistical analysis, three binary variables were constructed. Dichotomisation was chosen to permit logistic regression modelling and to ensure adequate cell counts given the available sample size; although binarising these variables reduces granularity, it improves the interpretability of the resulting odds ratios for a general medical education readership.

Place of origin was recoded from four categories (“large city,” “small city,” “suburban area,” and “rural area”) into a binary variable: urban environment (large city, suburban area) and rural environment (small city, rural area). In Slovenian context, the ‘large city’ response option referred to the country’s major urban centres and was therefore classified as urban, together with their suburban areas. The ‘small city’ category referred to smaller towns that often function as local service centres for the surrounding rural communities, including the provision of primary healthcare. Owing to their size, culture, settlement pattern, and close functional links with nearby rural areas, these towns were classified together with rural areas for the purposes of this analysis.

Experience with rural medicine was dichotomised into “no experience” (no involvement in rural clinical practice) and “has experience” (involvement for less than one week or more than one week).

### Statistical analysis

All statistical analyses were performed using RStudio. Two multivariate logistic regression models were constructed to identify independent predictors of interest in rural work and interest in family medicine specialisation, controlling for gender, university, home area, study year, and parental education level. Predictor variables were entered simultaneously, and odds ratios with 95% confidence intervals were calculated. Model fit was assessed and was found to be acceptable.

Likert-scale perception scores were treated as ordinal data. Group differences in perception scores between students with and without rural clinical experience were examined using the Wilcoxon rank-sum test (Mann-Whitney U test).

For binary categorical outcomes such as interest in rural practice and interest in family medicine, associations with rural experience were assessed using Pearson’s chi-square (χ²) test of independence. Where expected cell counts fell below five, Fisher’s exact test was applied.

The internal consistency of the 14-item Likert perception scale was evaluated using Cronbach’s alpha (α). The scale demonstrated acceptable internal consistency (α = 0.695), consistent with thresholds considered appropriate for exploratory research in medical education. Because Cronbach’s α reflects reliability rather than validity, it should be interpreted as a measure of internal consistency rather than of construct validity. No imputation was performed for missing item responses. Participants with incomplete questionnaires were excluded per the pre-specified inclusion criteria. A two-tailed p-value of < 0.05 was used as the threshold for statistical significance throughout.

### Ethical considerations

The study was conducted in accordance with the principles of the Declaration of Helsinki and applicable national guidelines for research involving human participants. Ethical approval was granted by the National Medical Ethics Committee of the Republic of Slovenia on 21 May 2024 (approval number 0120–125/2024-2711-7).

## Results

### Demographic characteristics

A total of 329 medical students participated in the study (response rate 19.1%). Table 1 represents a summary of the demographic characteristics of students involved in this study.

**Table 1:** Demographic data

Category	*N* (%)
University	
Ljubljana	234 (71.3%)
Maribor	94 (28.7%)
Study Year	
1st year	72 (22%)
2nd year	54 (16.2%)
3rd year	56 (17.1%)
4th year	76 (23.2%)
5th year	38 (11.6%)
6th year	24 (7.3%)
Gap year	9 (2.7%)
Gender	
M	79 (24.1%)
F	248 (75.3%)
other	1 (0.3%)
undefined	1 (0.3%)
Home Area	
Urban	128 (39%)
Rural	201 (61%)
Father’s Education Level	
Elementary school	3 (0.9%)
High school	98 (29.9%)
Associate degree	37 (11.3%)
Bachelors degree or higher	191 (57.9%)
Mother’s Education Level	
Elementary school	6 (1.8%)
High school	55 (16.8%)
Associate degree	38 (11.3%)
Bachelors degree or higher	230 (70.1%)

Home area differed by university: 137/234 (58.5%) of UL MF students reported a rural background, compared with 63/94 (67.0%) of UM MF students; this difference was not statistically significant (χ² *p* = 0.194). As an additional check for representativeness, respondents were divided into the first and last quartiles by questionnaire submission date (*n* = 82 each) and compared across all demographic variables. The two groups did not differ significantly in university, sex, home area, father’s education, mother’s education, or prior rural clinical experience (all χ² *p* ≥ 0.35). Study year distribution differed significantly between quartiles (χ² *p* = 0.035), with early respondents relatively over-represented in years 2 and 4 and late respondents over-represented in years 1 and 3.

### Experience in rural medicine

Overall, students reported rural clinical experience during medical training, with notable differences between universities. A minority of students (122; 37.1%) had rural clinical experience, whereas the majority did not (206; 62.9%). At the University of Ljubljana, 108 (46%) of students had rural clinical experience and 127 (54%) had none. At the University of Maribor, rural clinical experience was reported by 14 (14.9%) of students, whereas 80 (85.1%) reported no such experience. Table 2 provides an overview of the study years in which students from each university reported rural clinical experience.

**Table 2:** Rural clinical experience by year

University	*N* (%)
Ljubljana	
Year 1	80 (40.4%)
Year 2	45 (22.7%)
Year 3	37 (18.7%)
Year 4	17 (8.6%)
Year 5	7 (3.5%)
Year 6	12 (6.1%)
Maribor	
Year 1	6 (22.2%)
Year 2	3 (11.1%)
Year 3	4 (14.8%)
Year 4	6 (22.2%)
Year 5	3 (11.1%)
Year 6	5 (18.5%)

### Understanding of rural work environment

**Fig. 2:**
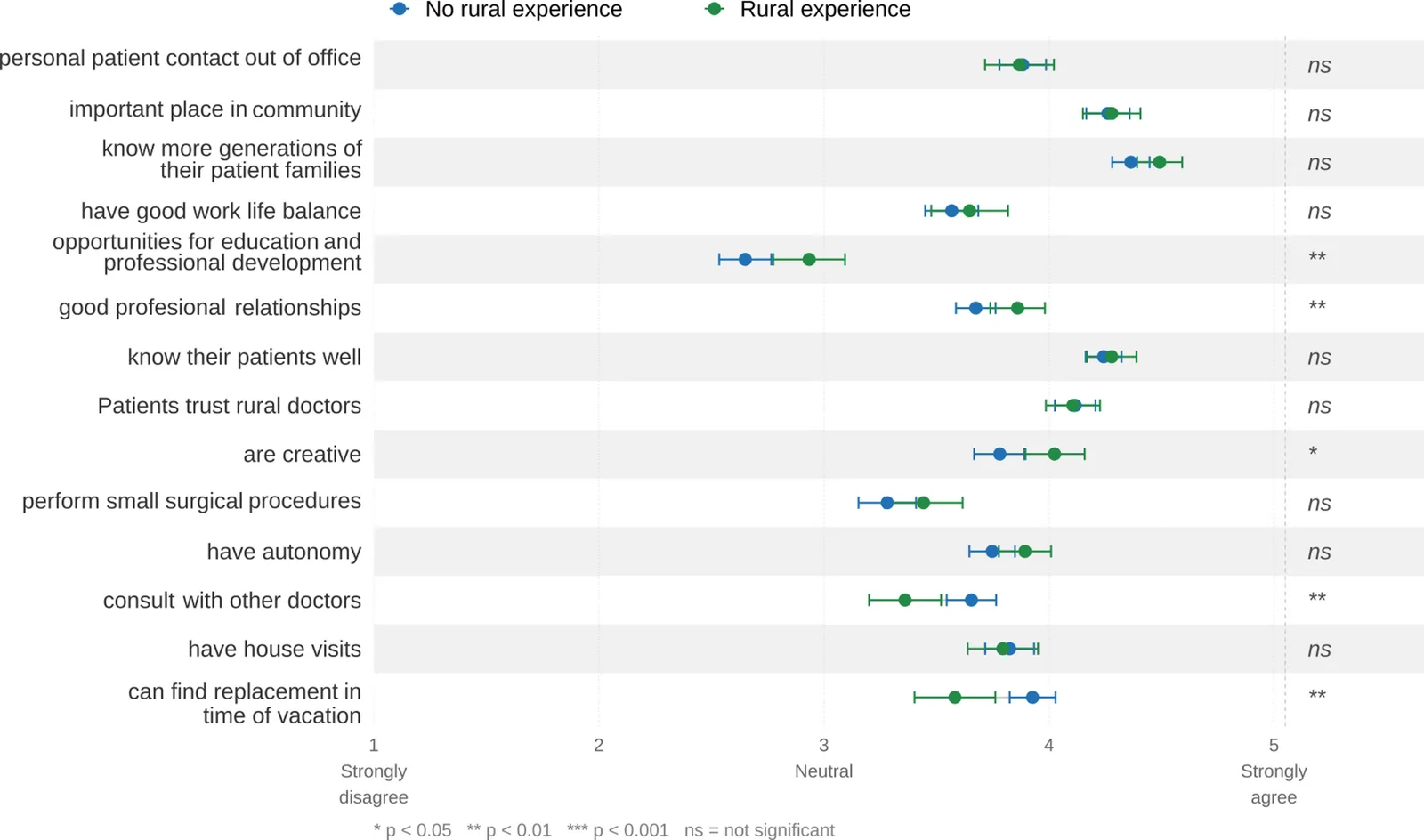
Perceptions of rural work environment among medical students with and without rural clinical experience. Mean Likert scores (1–5) for perceptions of rural doctors, compared between students with and without rural clinical experience. Points represent group means; horizontal bars indicate 95% confidence intervals. Statements are ordered by the difference in means (rural clinical experience minus no experience). Statistical comparisons were performed using the Wilcoxon rank-sum test. * *p* < 0.05, ** *p* < 0.01, *** *p* < 0.001, ns = not significant

Figure 2 presents mean Likert scores (1–5) for students’ perceptions of rural medical practice, stratified by prior rural clinical experience. Students reported generally positive perceptions across most statements, with mean scores predominantly above the neutral midpoint.

Students with rural clinical experience demonstrated significantly more favourable perceptions in several domains. Specifically, they reported higher agreement that rural doctors have good professional relationships (Fig. 2, *p* < 0.01), are creative (Fig. 2, *p* < 0.05), and have opportunities for education and professional development (Fig. 2, *p* < 0.01). Additionally, students with experience were more likely to agree that rural doctors can consult with other doctors (Fig. 2, *p* < 0.01). Conversely, students without rural clinical experience reported higher agreement that rural doctors can find a replacement during vacation (Fig. 2, *p* < 0.01). No statistically significant differences were observed between groups in several domains, including perceptions of autonomy, work–life balance, patient trust, community role, personal patient contact, and the performance of small surgical procedures (Fig. 2, all *p* > 0.05). Both groups consistently rated statements related to patient relationships and community importance highly.

### Interest in family medicine and rural medicine

**Fig. 3:**
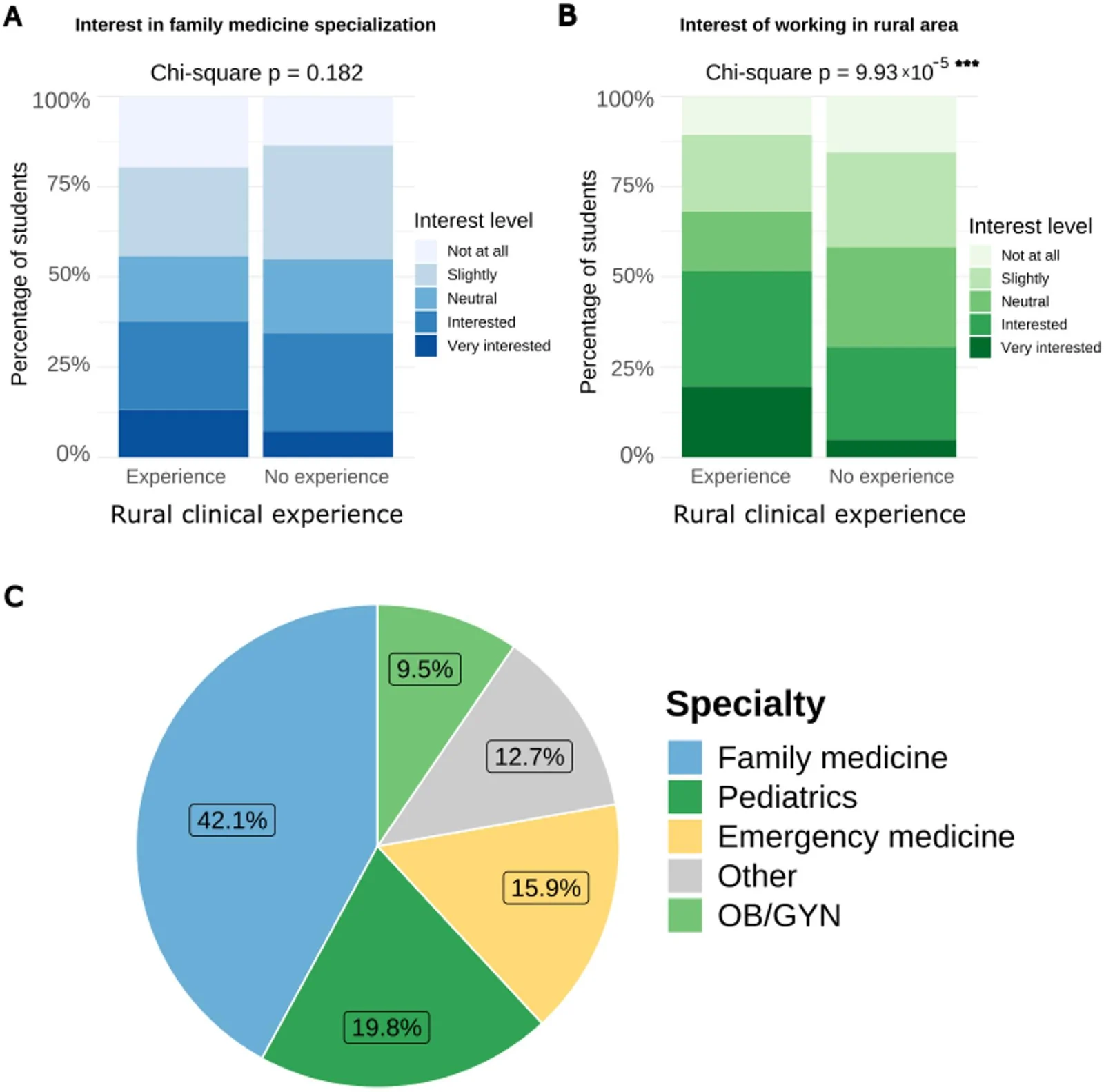
Student interest in family medicine, rural work, and preferred rural specialty. (**A**) Distribution of interest in specialising in family medicine among students with and without rural clinical experience. No significant difference was observed between groups (χ² *p* = 0.182). (**B**) Distribution of interest in working in a rural area among students with and without rural clinical experience. Students with rural clinical experience reported substantially higher interest than those without experience (χ² *p* = 9.93 × 10⁻⁵ ***). (**C**) Preferred rural medical specialties among students expressing interest in rural work

For family medicine specialisation, 15.9% of students reported no interest, 29.0% slight interest, 19.5% were neutral, 26.2% were interested, and 9.5% were very interested. Interest in rural work showed a similar distribution, with 13.7% reporting no interest, 24.4% slight interest, 23.5% neutral, 28.0% interested, and 10.4% very interested. Interest in specialising in family medicine did not differ between students with and without rural clinical experience (χ² *p* = 0.182; Fig. 3A). In contrast, interest in working in a rural area was significantly associated with rural clinical experience (χ² *p* < 0.001; Fig. 3B). Among students expressing interest in rural practice, family medicine was the most commonly chosen specialty (*N* = 53, 42.1%), followed by paediatrics (*N* = 25, 19.8%), emergency medicine (*N* = 20, 15.0%), other specialties (*N* = 16, 12.7%), and obstetrics/gynaecology (*N* = 12, 9.5%) (Fig. 3C). Students who selected “other” expressed interest in psychiatry, neurology, surgery, pathology, internal medicine, radiology and occupational medicine specialties.

Rural home background was the only statistically significant independent predictor of interest in family medicine specialisation, with students from a rural background having more than twice the odds of interest compared to urban students (OR 2.19, 95% CI 1.31–3.71, *p* = 0.003). Gender (OR 1.53, 95% CI 0.88–2.75, *p* = 0.142), university (OR 1.39, 95% CI 0.79–2.43, *p* = 0.248), study year (OR 1.10, 95% CI 0.95–1.29, *p* = 0.207), mother’s education (OR 1.28, 95% CI 0.72–2.29, *p* = 0.411), father’s education (OR 1.26, 95% CI 0.73–2.19, *p* = 0.411), and rural clinical experience (OR 1.01, 95% CI 0.57–1.75, *p* = 0.986) did not reach statistical significance. The model demonstrated acceptable fit.

Two predictors were independently and statistically significantly associated with interest in working in a rural area. Students with any prior rural clinical experience had nearly three times higher odds of expressing interest in rural work compared to those with no experience (OR 2.87, 95% CI 1.64–5.09, *p* < 0.001). Students from a rural background also had significantly higher odds of interest compared to those from urban areas (OR 1.85, 95% CI 1.11–3.11, *p* = 0.019). Gender (OR 1.08, 95% CI 0.62–1.90, *p* = 0.782), university (OR 1.37, 95% CI 0.78–2.44, *p* = 0.275), study year (OR 0.87, 95% CI 0.74–1.02, *p* = 0.085), mother’s education (OR 1.16, 95% CI 0.66–2.07, *p* = 0.615), and father’s education (OR 0.83, 95% CI 0.48–1.43, *p* = 0.497) were not independently associated with rural work interest. The model demonstrated acceptable fit.

### Motivating factors for work in rural medicine

**Fig. 4:**
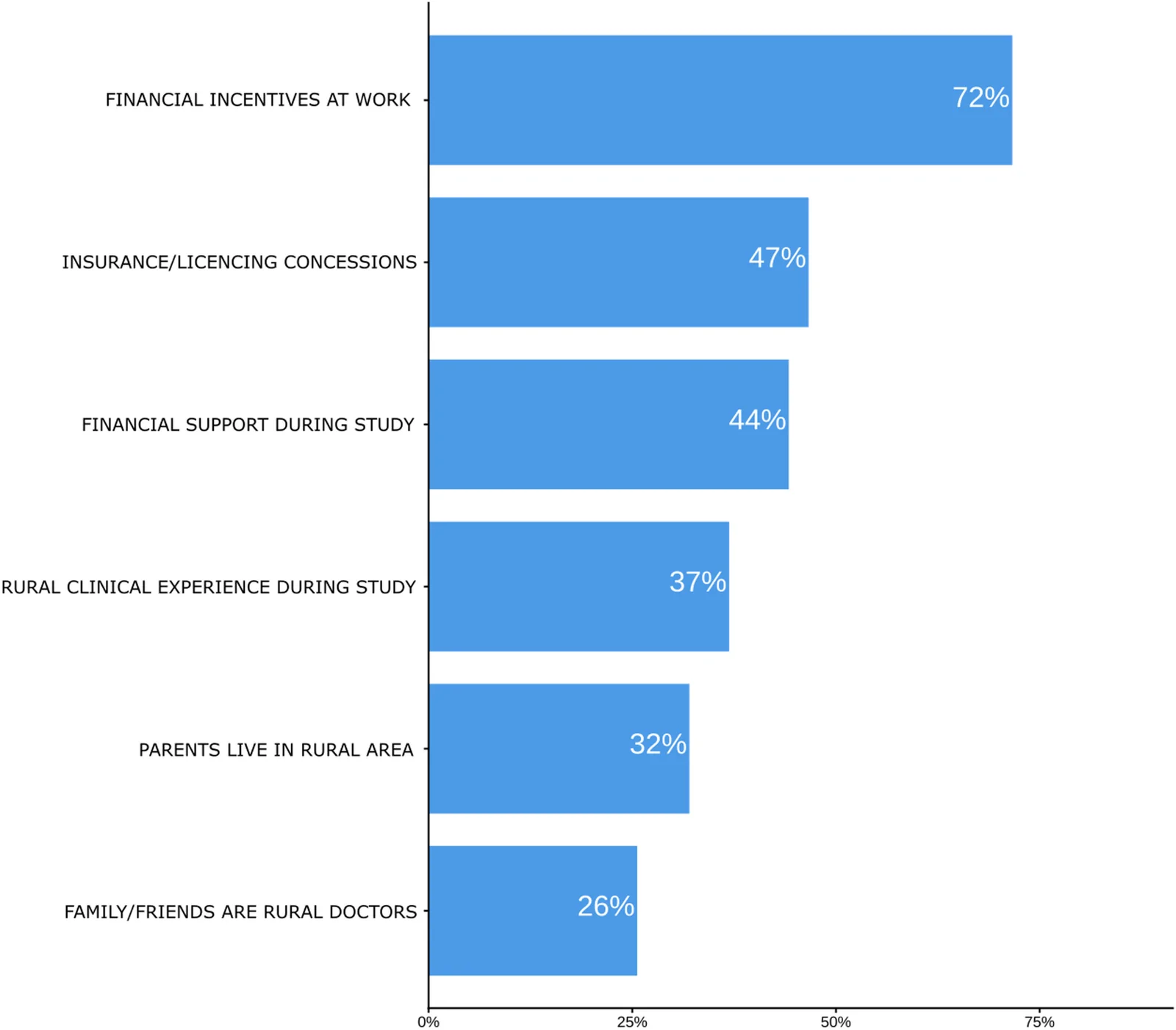
Factors impacting students’ choice for working in rural medicine

Figure 4 shows the proportion of students who selected different factors that would encourage them to work in a rural area. Financial incentives at work were most commonly selected (*N* = 235, 72%), followed by insurance or licensing concessions (*N* = 153, 47%) and financial support during study (*N* = 145, 44%). 37% (*N* = 121) of students indicated that rural clinical experience during study influenced their interest. Personal or social factors were selected less frequently. Only 32% (*N* = 105) of students reported that having parents living in a rural area would motivate them, and 26% (*N* = 84) felt influenced by family or friends working as rural doctors. Overall, rural clinical experience was consistently associated with more positive perceptions and greater interest in rural practice.

## Discussion

This study examined Slovenian medical students’ perceptions of rural medical practice, their interest in rural careers, and the motivational factors shaping those career intentions. Three principal findings emerge. First, prior rural clinical experience and rural home background are significantly associated with more favourable perceptions of rural practice and with greater interest in working in rural areas. Second, the two medical faculties differ substantially in the rates of medical students’ rural clinical experience, a difference that mirrors their curricular discrepancies. Third, financial and structural incentives are key motivators for rural career choice.

### Interest in working rurally and pursuing a family medicine specialty

As reported above, rural home background was the sole independent predictor of interest in family medicine, whereas interest in rural work was independently predicted by both rural upbringing and prior rural clinical experience. The association between rural clinical experience and greater interest in rural careers is consistent with international evidence showing that rural background and early rural placements are robust predictors of rural practice intentions [12, 13, 15, 23–25].

The finding that rural clinical experience was significantly associated with interest in working rurally (χ² *p* < 0.001) but not with interest in specialising in family medicine (χ² *p* = 0.182) is particularly noteworthy. It suggests that rural placements may shape a student’s willingness to practise in rural settings independently of specialty preference, and that the decision to pursue family medicine is determined by other factors. Prior literature shows that factors associated with interest in family medicine include female gender, older age at enrolment, rural upbringing, a preference for continuity of care, and desire for a shorter residency pathway [26].

This pattern appears to reflect a broader regional challenge, with neighbouring Hungarian medical students reporting low interest in family medicine specialty and rural practice [21], while Croatian medical students have interest in rural work, but feel insufficiently prepared to practise in rural settings [27]. To address this, Italy has implemented structured mentorship models, pairing rural-placement trainees with an experienced tutor who reviews their clinical encounters and provides real-time feedback on decision-making [28]. In contrast, a high proportion of Austrian medical students and residents (70–80%) were interested in working in rural areas [29]. The comparatively high interest reported in Austria should be interpreted with caution, as it derives from a single-institution study rather than a nationally representative sample. Nevertheless, Austria has established rural training pathways, financial incentives for rural practice, and regional workforce planning mechanisms that may make rural careers more attractive [30]. These structural characteristics may partly explain the higher reported interest, underscoring that rural career intentions are influenced not only by students’ experiences but also by the broader organisation and incentive structures of the healthcare system. These regional contrasts show that specialty choice and rural career intentions are shaped differently across health‑system contexts.

However, the strength and nature of our associations may be context-dependent, influenced by local healthcare system structure, educational pathways, and the organisation of rural practice. In the Slovenian context, where geographic distances are relatively small and healthcare infrastructure is more uniformly distributed, the mechanisms underlying this relationship may differ from those observed in larger or more ruralised countries. Given the cross-sectional design and potential self-selection bias, it remains unclear whether rural clinical experience itself drives this interest or whether students already inclined toward rural careers are more likely to seek such experiences.

### Student perceptions of rural work

It has been shown that students with rural background or rural clinical experience have better perception of rural work [31, 32]. Sustained, longitudinal placements can reshape career trajectories by prompting reflection that reinforces or challenges pre-existing career intentions [32]. Across all students, irrespective of rural clinical experience, perceptions of rural doctors were broadly positive: students consistently agreed that rural physicians know their patients well, enjoy patient trust, hold an important community position, maintain good collegial relationships, and have a favourable work–life balance. Similar patterns have been reported in Germany, where students perceive rural work as demanding yet compatible with better work–life balance [31]. Students with rural clinical experience held significantly more favourable views on rural doctors’ professional relationships (*p* = 0.006), creativity (*p* = 0.011), opportunities for professional development (*p* = 0.005), and opportunities for consultation with other specialists (*p* = 0.005). The last finding is particularly important given that professional isolation is a well-documented concern in rural medicine and a barrier to recruitment [4, 12]. Students without experience may overestimate the degree of isolation, whereas those who have worked in rural settings gain a more nuanced understanding of peer networks and informal consultation structures.

These findings suggest that even brief rural placements are associated with more accurate perceptions of rural practice, countering misconceptions that might otherwise deter students from rural careers. Similarly brief conversations with rural physicians have been shown to be effective in inspiring and recruiting future rural workforce [15]. Rural clinical experience has positively influenced students’ perceptions of rural practice and the quality of these placements, particularly the presence of inspiring mentors and meaningful opportunities for clinical skill development, appear to be important factors [14]. Although our questionnaire did not assess the quality of rural placements in detail, a positive trend toward a better understanding of rural physicians’ work was observed among students with rural clinical experience.

The large and statistically significant difference in rural clinical experience rates between the two faculties directly reflects curricular differences. The threefold difference in experience rates is therefore not surprising, but it has significant implications for workforce equity: graduates of the two faculties enter the labour market with systematically different rural career orientations, a disparity that is an artefact of institutional design rather than student preference. Although curricular design is the most plausible explanation for the observed difference, it is unlikely to be the only factor. The two faculties may also vary in their institutional culture and in their emphasis on rural and family medicine, independent of formal curricular content. Admission to both programmes is merit-based and organised through a single national application system in which students rank their preferred programmes, Moreover, despite nationally coordinated admissions, differences in geographic catchment areas and in the proportion of students with a rural background could also contribute to the patterns we observed. In our sample, the proportion of students reporting a rural home background differed between UL MF (58.5%) and UM MF (67%), although this difference was not statistically significant (χ² *p* = 0.194). Disentangling the relative influence of curricular exposure, institutional culture, and self-selection is beyond the scope of this cross-sectional study.

### Preferred specialty and motivating factors

Previous research has shown that approximately 36% of Slovenian medical students are interested in family medicine specialty [18]. Our data showed that 35.7% and 38.5% of students were interested in family medicine and rural work respectively. Among students interested in rural work, family medicine was the most preferred specialty, followed by paediatrics and emergency medicine. This ordering reflects the actual structure of rural primary care in Slovenia, where family physicians are the cornerstone of community health, typically serving as both gatekeepers and continuity-of-care providers. The fact that paediatrics and emergency medicine rank second and third may reflect student perceptions of these specialties as particularly well-suited to the generalist, high-responsibility character of rural practice. Notably, students who selected “other” listed psychiatry, surgery, and internal medicine, suggesting that some students envision broader scope roles in rural settings. Similar trends have been reported among medical students in the United States, where students who were interested in rural practice were also interested in specialties such as emergency medicine, general surgery, family medicine, internal medicine, paediatrics, and orthopaedics [23]. Combined specialty pathways such as family medicine obstetrics model, which allows physicians to integrate full-scope maternity care into generalist rural practice, may be an efficient way to accommodate this specialty interest overlaps [33] .These patterns indicate that rural–urban work options for such specialties should be considered [14].

Financial incentives emerged as the predominant motivating factor for rural career choice, considerably outweighing licensing concessions, financial support during study, and rural clinical experience gained during training. Personal and social factors ranked lowest in relative importance. This hierarchy is consistent with international evidence indicating that financial incentives constitute among the most effective instruments for rural physician workforce recruitment [12, 23, 27, 34]. Educational interventions are most common recruitment strategy in Europe [34]. Introducing structured and potentially mandatory short-term rural placements during undergraduate training could represent a feasible, low-cost intervention to increase experience and interest in rural practice. In addition, targeted financial incentive programmes, such as rural scholarships, providing real estate, or bonded contracts requiring a period of rural service after graduation, may be effective in attracting graduates to underserved areas. Aligning curricular opportunities across both medical faculties, particularly by expanding access to rural electives and mentorship, could further reduce disparities in rural career orientation and contribute to a more balanced distribution of the future medical workforce.

### Strengths and limitations

Despite these limitations, this study makes an original contribution by providing the first nationally representative data on Slovenian medical students’ perceptions of and motivations toward rural practice. The findings offer actionable, evidence-based guidance for both medical faculties and healthcare policymakers seeking to address the growing rural physician shortage in Slovenia. Given the evidence demonstrating that educational interventions can positively shift student perceptions of rural practice [15], there is reason for optimism that targeted curricular reforms, even modest ones, can move the needle on rural workforce development in the Slovenian context.

Several limitations should be considered when interpreting these findings. The cross‑sectional design precludes causal inference, and students already inclined toward rural careers may have been more likely to seek rural placements, introducing self‑selection bias. The response rate of 19.1% is consistent with rates typically reported for web-based surveys of medical student populations including a comparable nationwide German survey of medical students, which reported a response rate of 14.1% [35] and falls within the 10–30% range commonly observed in online surveys [36]. Nonetheless, this response rate raises the possibility of non-response bias. Respondents may differ systematically from non-respondents, particularly if students with pre-existing interest in rural practice or prior rural experience were more motivated to participate. This could inflate estimates of rural career interest and strengthen the observed association between rural experience and rural intentions. As an indirect check for non-response bias, we compared early and late respondents (first versus last quartile by submission date), a standard proxy for respondent/non-respondent differences [37]. Place of origin was self-reported using broad categories and may not capture the full diversity of settlement types and could have resulted in some degree of misclassification. Place of origin was self-reported using broad categories, which may not fully capture settlement diversity and could introduce misclassification. Rural experience was also self-reported and varied widely in duration and structure, from single observational sessions to multi-week supervised electives, likely diluting the measurable effect of high-quality exposure. Because rural experience, perceptions, and career intentions were assessed simultaneously using a self‑administered questionnaire, measurement bias is also possible, including socially desirable responding among students aware of the study’s focus.

The 14‑item perception scale demonstrated acceptable internal consistency. However, the instrument was purpose‑built for this study, informed by qualitative analysis of student seminar papers and relevant literature, and pilot tested with ten students for clarity and structure. It has not undergone formal psychometric validation, limiting conclusions about construct validity and reducing comparability with studies using established tools. Future work should prioritise full validation before applying this instrument in other populations or settings. Finally, the uneven distribution of respondents between faculties reflects true enrolment patterns but reduces the power of faculty‑level comparisons.

## Conclusions

This study demonstrates that rural clinical experience during medical training and rural background are associated with more favourable perceptions of rural practice and a stronger interest in pursuing a rural career. Students from rural backgrounds were more inclined toward both rural work and family medicine, while rural work experience was additionally associated with rural career interest. Marked disparity in rural clinical experience between the two faculties reflects the difference in curriculum design rather than student preference. Financial and structural incentives were the strongest motivators for rural career choice, suggesting that existing national support for family medicine training could be extended to rural medicine programmes. Family medicine remained the most common specialty preference among students interested in rural work, reflecting its central role in the Slovenian rural healthcare system. These findings support coordinated action by medical schools and policymakers to expand structured rural placements, harmonise curricula, and strengthen targeted financial incentives.

## Supplementary Information

Supplementary Material 1.

## Data Availability

The datasets generated and analysed during the current study are not publicly available due to privacy considerations but are available from the corresponding authors on reasonable request.

## Abbreviations

CI: Confidence Interval
OR: Odds Ratio
UL MF: Faculty of Medicine, University of Ljubljana
UM MF: Faculty of Medicine, University of Maribor

## References

[1] Long JC, Delamater PL, Holmes GM. Which Definition of Rurality Should I Use? The Relative Performance of 8 Federal Rural Definitions in Identifying Rural-Urban Disparities. Med Care. 2021;59(Suppl 5):S413–9. 10.1097/MLR.0000000000001612. PMC8428861 34524237

[2] Medical Association A. Defining rural for the physician workforce | AMA. 2024. https://www.ama-assn.org/system/files/issue-brief-defining-rural.pdf. Accessed 28 Mar 2026.

[3] Schaffer D, Bennett A. Beyond rural: A time-based measure for urban, rural, remote, and geographically isolated response. J Clim Change Health. 2026;27:100616. 10.1016/J.JOCLIM.2025.100616. PMC13184500 42164949

[4] Petrovcic R. Defining rural, remote and isolated practices: The example of Slovenia. Family Med Prim Care Rev. 2016;18:391–3. 10.5114/FMPCR/59129.

[5] Slovenia - Agriculture and rural development - European Commission. https://agriculture.ec.europa.eu/cap-my-country/cap-strategic-plans/slovenia_en. Accessed 15 Apr 2026.

[6] Map of municipalities according to degree of urbanisation. 2025. https://www.stat.si/dokument/14280/Map%20of%20municipalities%20according%20to%20d%20egree%20of%20urbanisation%202025.pdf. Accessed 28 Mar 2026.

[7] Global Human Settlement - Degree of urbanisation - European Commission. https://human-settlement.emergency.copernicus.eu/CFS.php. Accessed 29 May 2026.

[8] Schiavina M, Melchiorri M, Pesaresi M, Politis P, Carneiro Freire, et al. GHSL Data Package 2023, Publications Office of the European Union, Luxembourg, 2023. 10.2760/098587.

[9] Prebivalstvo po naseljih, podrobni podatki, Slovenija, 1. januar 2021. https://www.stat.si/StatWeb/News/Index/9578. Accessed 16 Mar 2026.

[10] Primary care accessibility. https://zdravniki.sledilnik.org/en/analytics. Accessed 29 Apr 2026.

[11] Arredondo K, Bay K, Witte L, Touchett H, Sonnenfeld M, Paterson-Roberts A, et al. Rural Practice Made Attractive: A Scoping Review of Rural Primary Care Physician Recruitment and Retention Incentives. J Gen Intern Med. 2026;4:1–12. 10.1007/S11606-026-10218-8/FIGURES/3. PMC13083479 41588280

[12] Budhathoki SS, Zwanikken PAC, Pokharel PK, Scherpbier AJ. Factors influencing medical students’ motivation to practise in rural areas in low-income and middle-income countries: a systematic review. BMJ Open. 2017;7. 10.1136/BMJOPEN-2016-013501. PMC5337703 28232465

[13] Holst J. Increasing Rural Recruitment and Retention through Rural Exposure during Undergraduate Training: An Integrative Review. 10.3390/ijerph17176423 PMC7503328 32899356

[14] Ray RA, Young L, Lindsay D. Shaping medical student’s understanding of and approach to rural practice through the undergraduate years: A longitudinal study. BMC Med Educ. 2018;18:1–8. 10.1186/S12909-018-1229-8/TABLES/1. PMC6013947 29929521

[15] Perez G, Malhi R, Bressler K, Monaghan M, Johnston A. Challenging perceptions about rural practice using narratives: a living library approach in medical education. Front Med (Lausanne). 2024;11:1452932. 10.3389/FMED.2024.1452932/TEXT. PMC11667788 39720659

[16] Perez G, Groves A, Fehr L, Johnston A. Peer-led learning: a novel approach to promote rural healthcare interest among medical students. Front Med (Lausanne). 2025;12. 10.3389/FMED.2025.1566472/FULL. PMC12066776 40357284

[17] Slovenia taken to task for inadequate access to healthcare. The Slovenia Times. 2025. https://sloveniatimes.com/43341/slovenia-taken-to-task-for-inadequate-access-to-healthcare. Accessed 28 May 2026.

[18] Ster MP, Selic P. Intended Career Choice in Family Medicine in Slovenia: An Issue of Gender, Family Background or Empathic Attitudes. Final Year Med Students? Mater Sociomed. 2017;29:143. 10.5455/MSM.2017.29.143-148. PMC5544448 28883780

[19] Liu J, Zhu B, Mao Y. Association between rural clinical clerkship and medical students’ intentions to choose rural medical work after graduation: A cross-sectional study in western China. PLoS ONE. 2018;13:e0195266. 10.1371/JOURNAL.PONE.0195266. PMC5880380 29608624

[20] Geier AK, Heuser A, Bleckwenn M, Deutsch T. Rural doctor quota students in Germany–who are they? Data on first year students from two cohorts in the federal state of Saxony. Med Educ Online. 2025;30. 10.1080/10872981.2025.2497325. PMC12064117 40329552

[21] Mohos A, Szőllősi GJ, Kolozsvári LR, Rinfel J, Varga A, Kucsera MM, et al. Rural family medicine as a career option among Hungarian medical students. Eur J Gen Pract. 2023;29. 10.1080/13814788.2023.2174258. PMC9937003 36794681

[22] Zvrko E, Popović N, Radunović M, Nikolić G. Motivational Factors Influencing the Choice of Medical Studies and Future Career Plans among Montenegrin Students. Slovenian J Public Health. 2024;63:132–41. 10.2478/SJPH-2024-0018. PMC11178028 38881636

[23] Meade ZS, Li HW, Allison H, Bhatia MB, Joplin TS, Simon C, et al. Demographics and medical school exposures to rural health influence future practice. Surg (United States). 2022;172:1665–72. 10.1016/j.surg.2022.08.016. 36127171

[24] Woloschuk W, Tarrant M. Does a rural educational experience influence students’ likelihood of rural practice? Impact of student background and gender. Med Educ. 2002;36:241–7. 10.1046/J.1365-2923.2002.01143.X. 11879514

[25] Beattie J, Binder M, Fuller L. Rural longitudinal integrated clerkships and medical workforce outcomes: A scoping review. Med Teach. 2024;46:545–55. 10.1080/0142159X.2023.2260082. 37769044

[26] Gill H, Mcleod S, Duerksen K, Olga M, Mhsa S. Factors influencing medical students’ choice of family medicine Effects of rural versus urban background. Can Family Physician • Can Fam Physician. 2012;58:649–57. PMC3498039 23152472

[27] Mrduljaš-Ðujić N, Radić I, Bašić Marković N, Vrgoč T, Buljubašić M. Attitudes of medical students in Croatia toward rural medicine education and practice. Front Med (Lausanne). 2025;12:1485790. 10.3389/FMED.2025.1485790/TEXT. PMC11842319 39991060

[28] Cavicchi A, Venturini S, Petrazzuoli F, Buono N, Bonetti D. INFORMEG, a new evaluation system for family medicine trainees: feasibility in an Italian rural setting. Rural Remote Health. 2016;16. 10.22605/RRH3666. 27506900

[29] Mahlknecht A, Engl A, Barbieri V, Bachler H, Obwegeser A, Piccoliori G, et al. Attitudes towards career choice and general practice: a cross-sectional survey of medical students and residents in Tyrol, Austria. BMC Med Educ. 2024;24. 10.1186/s12909-024-05205-8. PMC10943776 38491385

[30] Simoens S. Experiences of Organization for Economic Cooperation and Development countries with recruiting and retaining physicians in rural areas. Aust J Rural Health. 2004;12:104–11. 10.1111/J.1440-1854.2004.00569.X. 15200520

[31] Brütting C, Herget S, Nafziger M, Klingenberg A, Deutsch T, Frese T, et al. Factors promoting willingness to practice medicine in rural regions and awareness of rural regions in the university’s catchment area – cross-sectional survey among medical students in central Germany. GMS J Med Educ. 2023;40. 10.3205/ZMA001634. PMC10407585 37560039

[32] Beattie J, Binder M, Beks H, Fuller L. Influence of a rural Longitudinal Integrated Clerkship on medical graduates’ geographic and specialty decisions: a constructivist grounded theory study. BMC Med Educ. 2024;24:795. 10.1186/S12909-024-05793-5/FIGURES/3. PMC11267796 39049054

[33] Fortenberry KT, Owens RW, Stoesser K, Adediran E, Whittaker TC, Kiraly B. Family Medicine Obstetrics Fellowship Funding and Characteristics. Fam Med. 2026;58:12–9. 10.22454/FAMMED.2026.646169. PMC12969530 41854848

[34] Bes JM, Flinterman LE, González AI, Batenburg RS. Recruitment and retention of general practitioners in European medical deserts: a systematic review. Rural Remote Health. 2023;23. 10.22605/RRH7477. 36854290

[35] Bauer J, Groneberg DA. Physicians’ working conditions in hospitals from the students’ perspective (iCEPT-Study)-Results of a web-based survey. J Occup Med Toxicol. 2016;11:5. 10.1186/S12995-016-0094-9/FIGURES/3. PMC4759714 26900392

[36] Wu MJ, Zhao K, Fils-Aime F. Response rates of online surveys in published research: A meta-analysis. Computers Hum Behav Rep. 2022;7:100206. 10.1016/J.CHBR.2022.100206.

[37] Armstrong JS, Overton TS. Estimating Nonresponse Bias in Mail Surveys. J Mark Res. 1977;14:396–402. 10.1177/002224377701400320.

