# Supplementary material for: Association between rural clinical experience and rural career intentions amongst Slovenian medical students: a national cross-sectional study
Source: BMC Med Educ. 2026 Aug 11;26:1797. doi: 10.1186/s12909-026-10133-w (PMC13741519; doi:10.1186/s12909-026-10133-w)
Supplement: Supplementary file 1 [file 12909_2026_10133_MOESM1_ESM.docx]

# SUPPLEMENTARY MATERIAL

**Study Questionnaire**

**ENGLISH TRANSLATION**

**SECTION 1 — DEMOGRAPHIC DATA**

Q1. Age: ___________

Q2. Year of study: 1st / 2nd / 3rd / 4th / 5th / 6th / Final year (absolvent)

Q3. Gender: Male / Female / Other / Prefer not to say

Q4. University: Ljubljana / Maribor

Q5. Home region (region where you spent most of your childhood):

Gorenjska / Goriška / Jugovzhodna Slovenija / Koroška / Obalno-kraška / Osrednjeslovenska / Podravska / Pomurska / Posavska / Primorsko-notranjska / Savinjska / Zasavska / Abroad

Q6. How would you describe your place of origin?

Large city (e.g., Ljubljana, Maribor, Kranj, Novo Mesto…) / Small city (e.g., Domžale, Vrhnika, Izola, Ptuj…) / Suburban area (e.g., Brezovica pri Ljubljani, Škofljica, Vodice…) / Rural area

Q7. Mother’s level of education:

Primary school / Secondary school / Higher vocational / University degree or higher

Q8. Father’s level of education:

Primary school / Secondary school / Higher vocational / University degree or higher

**EXPERIENCE WITH RURAL MEDICINE**

Q9. Experience with rural clinical practice:

(Clinics that are not part of a health centre in a major city, and that also cover very remote areas. Rural practice includes clinical work, volunteering, and other forms of involvement.)

- I have not been involved in rural clinical practice
- I have been involved in rural clinical practice for less than one week
- I have been involved in rural clinical practice for more than one week.

Q10. (If Q9 = options 2 or 3) In which year(s) of study were you involved in rural clinical practice? (Multiple answers possible): 1st / 2nd / 3rd / 4th / 5th / 6th

**FUTURE CAREER**

Q12. How likely is it that you will practise medicine in a rural setting in the future?

(1) Not at all likely / (2) Unlikely / (3) Very likely / (4) Certain

**PERCEPTIONS OF RURAL MEDICAL PRACTICE**

Q13. In the following, we are interested in your personal opinion. There are no right or wrong answers. Please rate each statement about the work of a rural doctor on a scale of 1–5, where 1 = strongly disagree and 5 = strongly agree.

1. Rural doctors frequently have contact with their patients outside the clinic.

2. Rural doctors hold an important position in the community.

3. Rural doctors know several generations of their patients’ families.

4. Rural doctors have good opportunities to balance family and professional life.

5. Rural doctors have many opportunities for further education and professional development.

6. Rural doctors have good relationships with their colleagues.

7. Rural doctors know their patients well.

8. Patients trust their rural doctor.

9. Rural doctors are creative and resourceful in their work.

10. Rural doctors frequently perform minor surgical procedures.

11. Rural doctors have a high degree of professional autonomy in their work.

12. Rural doctors have many opportunities for consultation with other clinical specialists. [reverse-coded]

13. Rural doctors frequently conduct home visits.

14. Rural doctors find it difficult to arrange cover for absences during training or annual leave.

**CAREER PREFERENCES AND MOTIVATIONAL FACTORS**

Q14. Are you interested in specialising in family medicine?

(1) Not at all interested / (2) Not interested / (3) Neither interested nor uninterested / (4) Interested / (5) Very interested

Q15. Are you interested in working in a rural area?

(1) Not at all interested / (2) Not interested / (3) Neither interested nor uninterested / (4) Interested / (5) Very interested

Q16. (If Q17 = 4 or 5) If you are interested in working in a rural area, in which field would you like to work?

Family medicine / Gynaecology and obstetrics / Paediatrics / Emergency medicine / Other

Q17. Which factors would encourage you to work in a rural area? (Multiple answers possible):

- Financial incentives during study (scholarship/bursary)
- Financial incentives at work (official housing, additional pay, etc.)
- Possibility of obtaining a licence concession
- More exposure to rural medicine during study
- Parents living in a rural area
- Family or friends working as rural doctors
- Other

Q18. The number of available training posts for the specialties I am interested in will influence my choice of specialty.

(1) Strongly disagree / (2) Disagree / (3) Neither agree nor disagree / (4) Agree / (5) Strongly agree

Q19. Is there anything else you would like to tell us? We welcome your comments and suggestions.
